# Re-analysis on the statistical sampling biases of a mask promotion trial in Bangladesh: a statistical replication

**DOI:** 10.1186/s13063-022-06704-z

**Published:** 2022-09-15

**Authors:** Maria Chikina, Wesley Pegden, Benjamin Recht

**Affiliations:** 1 Department of Computational and Systems Biology, University of Pittsburgh, Pittsburgh, PA USA; 2 Department of Mathematics, Carnegie Mellon University, Pittsburgh, PA USA; 3 Department of Electrical Engineering and Computer Sciences, University of California, Berkeley, CA USA

## Abstract

A recent randomized trial evaluated the impact of mask promotion on COVID-19-related outcomes. We find that staff behavior in both unblinded and supposedly blinded steps caused large and statistically significant imbalances in population sizes. These denominator differences constitute the rate differences observed in the trial, complicating inferences of causality.

## Background

The efficacy and value of non-pharmaceutical interventions for mitigating COVID-19 remain hotly contested. Unfortunately, the scientific community performed very few randomized trials to establish evidence bases for allocating resources and minimizing harm in pandemic response plans. One notable exception was a large-scale cluster randomized controlled trial of mask promotion in Bangladesh, recently published in *Science* [1]. Since high impact publications often lead to changes in social behaviors and government policies, they need to be carefully vetted.

The Bangladesh mask trial reported decreases in symptomatic seroprevalence (primary outcome), decreases in reported COVID-19-like symptoms (secondary outcome), and increases in mask wearing behavior (secondary outcome). The study analyzed the data with a generalized linear model and found a 10% decrease in the primary endpoint, evaluating this result as significant at $p=0.05$.

**Fig. 1:**
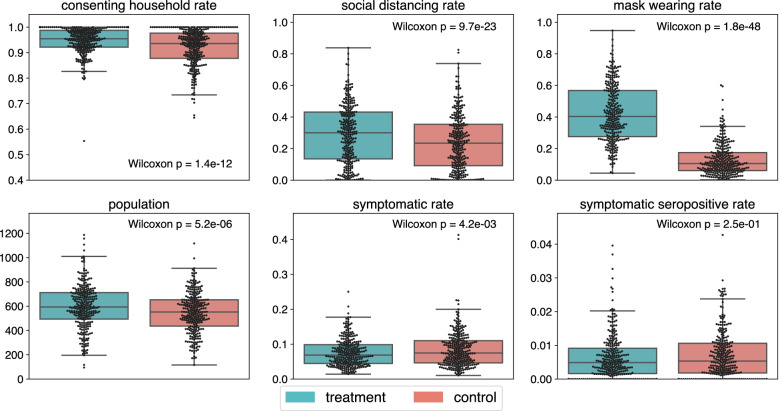
Differences between treatment and control groups. Each dot represents one village. Significance is evaluated with Wilcoxon paired tests, applied to the study-assigned pairs of treatment/control villages

In this commentary, we re-analyze this trial using simple non-parametric tests. Upon reanalysis, we find a large, statistically significant imbalance in the size of the treatment and control arms evincing substantial post-randomization ascertainment bias by unblinded staff. The observed decrease in the primary outcome is the same magnitude as the population imbalance but fails significance by the same tests (see Fig. 1 and Table 1). This reanalysis thus complicates drawing any causal link between masks and the observed decrease in population-rate of symptomatic seropositivity.

**Table 1:** Differences in treatment vs control groups. $\Delta$ denotes the percent difference between treatment and control. *p* is the *p*-values when significance is evaluated with Wilcoxon paired tests

	Intervention effect		Intervention effect for		Intervention effect for	
			Surgical masks		Cloth masks	
	$\Delta$	*p*	$\Delta$	*p*	$\Delta$	*p*
Mapped households						
Number	4.5%	7.2e−03	5.4%	5.6e−03	2.9%	4.5e−01
Reached households						
Fraction	2.8%	1.6e−12	2.6%	1.4e−07	3.1%	1.6e−06
Households consented						
Fraction	0.13%	1.2e−01	0.16%	1.2e−01	0.06%	7.3e−01
Consenting population						
Size	8.6%	5.2e−06	9.5%	4.8e−05	6.9%	2.5e−02
Sympto. seropositive						
Rate	− 8.7%	2.4e−01	− 9.4%	3.8e−01	− 7.0%	4.6e−01
Count	− 1.8%	9.7e−01	− 2.3%	1.0e00	− 6.0%	9.3e−01
Symptomatic						
Rate	− 10%	4.2e−03	− 11%	1.7e−02	− 8.3%	1.1e−01
Count	− 3.8%	4.2e−01	− 3.7%	4.3e−01	− 3.9%	7.1e−01
Social distancing						
Rate	22%	2.2e−16	25%	4.4e−18	17%	2.6e−06
Mask wearing						
Rate	220%	1.8e−48	240%	6.6e−33	190%	3.0e−17

Although raw numbers were not presented in the published paper, the primary outcome differed by a total of just 20 cases between the treatment and control arms: In a study population of over 300,000 individuals, there were 1106 symptomatic seropositives in control and 1086 in treatment. In particular, the difference in rates is constituted by denominator differences, and thus is similar in magnitude (10% vs 9%) to the population imbalance which arose through the interaction of staff bias and random chance (156,938 and 170,497 individuals enrolled in control and treatment respectively).

## Study protocol

In the study, 600 villages in Bangladesh were paired based on COVID-case data, population density, and population size. Each paired village was assigned to treatment or control at random. Households were enrolled in a two-step process. In the first step, blinded staff members mapped villages and household locations. In the second step, unblinded staff members sought consent from eligible household members; unblinded staff recorded for each household that they (a) consented to participate, (b) declined to participate, or (c) were “unreachable.” The study then proceeded to implement a mask promotion intervention in the treatment villages. No placebo intervention was implemented in control villages. All participants were asked to report COVID-like symptoms. Those who reported symptoms were asked to volunteer blood draws for serology. The primary endpoint was evaluated based on the fraction of the volunteered blood draws that tested positive for COVID antibodies. 14 village pairs were dropped from the study because of lack of government cooperation or sufficient observation, leaving a total of 286 pairs of villages in the final analysis. Upon publication of their results in *Science*, the authors made their data available in a gitlab repository^1^, and all of the quantities discussed in this paper are derived from the data in this repository.

## Impact of trial intervention

Both steps of enrollment contribute to the above noted 9% population imbalance. 4.5% more households were mapped in treatment villages than control villages in the first step. This difference in mapping behavior between treatment and control groups is significant with $p=0.0072$, despite supposed staff blinding.

In the second step, unblinded staff obtained consent from more and larger households, increasing the final imbalance in the number of households between treatment and control groups to 8%, and the imbalance in populations to 9%. Households were recorded absent 1.4× more frequently and having no eligible household member present 2.2× more frequently in the control arm.

To evaluate the impact of this bias, we tested many of the reported outcomes in the study for significance with paired Wilcoxon signed-rank tests at the village level. In these tests, the only randomness in observation assumed for test validity is the independent random choice of a control and treatment village from each village pair. In addition to the effects of the trial’s intervention on rates of mask-wearing and physical distancing, the difference in the consent obtained by unblinded staff is among the most significant differences of any outcome difference between treatment and control. Of the outcomes we tested with Wilcoxon tests, only these three had *p*-values less than $10^{-6}$ (see Fig. 1 and Table 1). In the second step of enrollment, staff was tasked with both enrolling households and providing masks in the treatment villages and hence were aware whether they were surveying a treatment or control village.

As described by the authors, this lack of staff blinding led to substantial post-randomization ascertainment bias (supplement, page 41 and Table S19). They suggest that the arm imbalance could be attributed to surveyors being more eager to enroll borderline households in the treatment villages and households volunteering individuals younger than 18 so that they could receive masks. However, in their robustness analyses of the imbalance, they are only able to account for 25% of the difference in size between treatment and control. The statistically significant differences in the supposedly blinded mapping step suggests that some unintentional unblinding may have also occurred.

Inferring causal effects in the presence of the strong effect on enrollment rates to which the findings are not robust requires assuming that borderline participants who would have been consented to participate in treatment but not control were just as likely as typical villagers to become infected with COVID, develop symptoms, and report them to study staff. More importantly, as blood draws were conditioned on symptom reporting and no bias resistant endpoints were evaluated, the substantial, highly significant effects on staff and participant behavior should caution against confident causal claims about COVID-related outcomes.

**Fig. 2:**
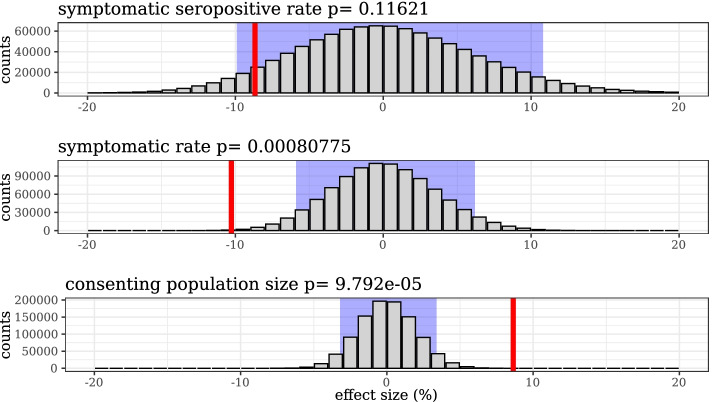
Empirical distribution of random variation in apparent effect sizes for symptomatic seropositivity rate, for symptom rate, and for population size, under simulated random treatment/control assignments. The *x*-axis range is the same across all three histograms. Consenting population is subject to considerably less variance from randomization than the other outcomes. The histograms are generated by reassigning treatment/control within village pairs randomly, 1 million times, to generate 1 million alternative splits between treatment and control groups. The red line shows the effect size for the actual treatment/control group split. Light blue rectangles contain 95% of the data

The $9\%$ imbalance observed in population sizes arose through some combination of bias and random chance. We ran permutation tests to illustrate the relative contribution each of these factors on the imbalance. Figure 2 displays histograms generated by reassigning treatment/control within village pairs randomly, 1 million times, to generate 1 million alternative splits between treatment and control groups. The red line shows the effect size for the actual treatment/control group split. We see that it would be exceptionally rare for the population imbalance to occur by random assignment of pairs to treatment and control. On the other hand, the observed symptomatic rates and symptomatic seropositivity rates are more plausibly explained by random fluctuation. That is, the null hypothesis that the intervention had no effect on the two outcomes based on subjective surveys is more likely than the the null hypothesis that the intervention had no effect on the population imbalance. We would expect the symptomatic rates and symptomatic seropositivity rates, which are based on subjective surveys, to be more susceptible to staff and participant bias than demographic quantities like “counts of households” and “number of people in a village.” In other words, to infer strong causal effects of the intervention on the COVID-related outcomes, the bias and randomization that imbalanced enrollment population—which is definitely not a direct causal effect of mask filtration—should be at least as likely to induce similar imbalances in COVID-related outcomes.

The authors provided similar permutation tests in Appendix Fig. S2 of their paper. Here, rather than simply counting the number of seropositives in each resampling, the authors re-run their fixed-effect regression to estimate the magnitude of the masking effect. They report the one-sided *p*-value of 0.07 for symptomatic seropositivity. The two-sided *p*-value associated with the authors’ permutation test is 0.14, which is aligned with our findings. We highlight this point to note that when we examine the same outcomes as the authors, the *p*-values in our reanalysis are not far from those reported in the original paper. However, we found other effects that should have been non-causal that were much more highly significant, suggesting that it is difficult to disentangle the effects of differences in staff-participant interaction between groups from the direct causal effects of masks.

**Fig. 3:**
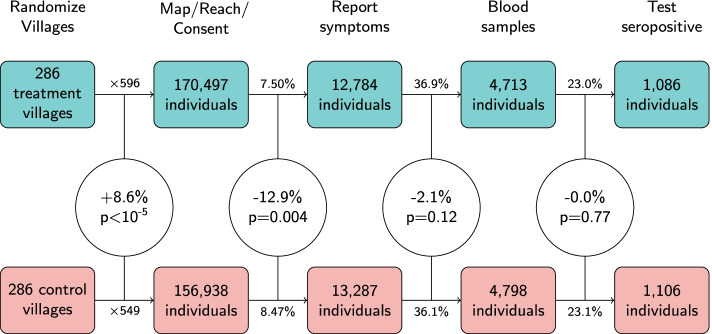
Steps leading to the final 1086:1106 split in symptomatic seroprevalence between treatment and control groups. Each circle shows how much greater or lower the transition rate is in the treatment group vs the control group (e.g., in the second step, 7.5% is 12.0% less than 8.5%), along with the statistical significance of this difference in Wilcoxon signed rank tests applied to the data at the level of treatment/control village pairs. We begin with 286 villages (rather than 300) because data was not released for 14 of the 300 village pairs

Figure 3 illustrates the steps leading to the final 1086:1106 split in symptomatic seroprevalence between treatment and control groups. Each circle shows how much greater or lower the transition rate is in the treatment group vs the control. The magnitude of these differences are striking: in behavioral outcomes, differences on the order of 10% were observed between the study arms. However, the same percentage of symptomatic individuals in consented to blood draws in both arms. Additionally, in the final step when blood samples are tested, there is no difference in the rate with which samples test positive for COVID-19 antibodies. It might seem surprising that the intervention’s impact on other behavioral mitigation measures such as social distancing also did not result in clear impact on symptomatic seropositivity.

## Conclusion

As it would not be reasonable to conclude from this trial that there is a direct causal link between mask wearing and the number of residents in villages and households, any causal claims based on effects of similar size in this trial should be considered with caution. In particular, both COVID symptoms and COVID symptomatic seroprevalence exhibited similar magnitudes (and much weaker significance) than population differences which arose from bias and chance alone.

It is tempting to argue that the recruited and unrecruited individuals would have to be substantially different in how they report symptoms or test positive on serology in order for the recruitment bias to entirely account for the reported effect on endpoints. While a large difference between these populations may be unlikely, the bias evident in the trial outcomes demands caution regardless of assumptions one might be willing to make about unobserved individuals in the control arm.

In particular, it is critical to consider that all of the outcomes in the study are based on self-reporting of symptoms. Even for the serology endpoint, which may appear unbiased at first glance, subjects were only eligible for a blood draw if they had reported symptoms. Thus, all endpoints are subject to behavioral biases. Our analysis of the population size shows that behavioral biases can produce a highly significant 9% difference between the control and intervention arm in the absence of any causal link with the intervention. It is thus also premature to conclude a similarly sized causal effect on *any other* variable that is subject to behavior bias, including the trial endpoints.

The purpose of randomized control trials is to establish a causal link between interventions and outcomes. However, causal implications are diminished in the presence of unblinding, ascertainment bias, and bias-susceptible endpoints. Unfortunately, in the Bangladesh mask trial we evidence of all of the above.

The study in question raises intriguing questions about the role of public health interventions in changing behavioral patterns to decrease COVID case rates in low- and middle-income countries. The mask intervention was highly effective at modifying behaviors (distancing, mask-wearing, symptom reporting). Nonetheless, the data is consistent with mask wearing having modest or no direct effect on COVID-related outcomes in this experimental setting.

## Data Availability

Code to reproduce our figures is available at https://github.com/mchikina/maskRCTnote

## Footnotes

1 https://gitlab.com/emily-crawford/bd-mask-rct
